# Modified Two-Stage Split Technique for Controlled Ridge Augmentation in Horizontally Atrophic Posterior Mandible: the First Stage of Research

**DOI:** 10.17691/stm2020.12.4.05

**Published:** 2020-08-27

**Authors:** A.I. Korsakova, I.A. Zhadobova, A.S. Klochkov, S.A. Durnovo, A.V. Kochubeynik, E.A. Durnovo

**Affiliations:** Tutor, Department of Oral and Maxillofacial Surgery with the Course of Plastic Surgery; Privolzhsky Research Medical University, 10/1 Minin and Pozharsky Square, Nizhny Novgorod, 603005, Russia; Tutor, Department of Oral and Maxillofacial Surgery with the Course of Plastic Surgery; Privolzhsky Research Medical University, 10/1 Minin and Pozharsky Square, Nizhny Novgorod, 603005, Russia; Associate Professor, Department of Oral and Maxillofacial Surgery with the Course of Plastic Surgery; Privolzhsky Research Medical University, 10/1 Minin and Pozharsky Square, Nizhny Novgorod, 603005, Russia; Student, Faculty of Dentistry; Privolzhsky Research Medical University, 10/1 Minin and Pozharsky Square, Nizhny Novgorod, 603005, Russia; Associate Professor, Academic Director of the Institute of Dentistry; Head of the Department of Propaedeutic Dentistry; Privolzhsky Research Medical University, 10/1 Minin and Pozharsky Square, Nizhny Novgorod, 603005, Russia; Professor, Director of the Institute of Dentistry; Head of the Department of Oral and Maxillofacial Surgery with the Course of Plastic Surgery Privolzhsky Research Medical University, 10/1 Minin and Pozharsky Square, Nizhny Novgorod, 603005, Russia

**Keywords:** jaw reconstruction, bone grafting, bone regeneration, horizontal atrophy of the jaws, alveolar ridge splitting, split-crest technique.

## Abstract

Various bone grafting methods are applied to eliminate horizontal atrophy of the jaws. However, problem complexity brings about ongoing research and development of new ways to achieve the predicted stable and long-term results of implantological treatment.

**The aim of the study** was to evaluate the results of the developed method for bone grafting, a modified two-stage split technique for controlled ridge augmentation in horizontally atrophic posterior mandible, using radiological analysis data.

**Materials and Methods.:**

The study group included 18 patients with horizontally atrophic posterior mandible. According to cone beam computed tomography, 39 jaw segments were assessed before plastic surgery and after applying the two-stage split-crest technique for controlled ridge augmentation. The alveolar ridge width was estimated in the area of its top and at a distance of 1, 3, 5 mm from it using the vector of future implant position and taking into account the angle of inclination of the atrophic region of the mandible.

**Results.:**

When analyzing edentulous areas in the posterior mandible before treatment, there was rather a large angle of lingual inclination of the alveolar ridge. After 6 months, the average increase in bone tissue width in the region of the alveolar ridge top was 82%, it was 50.6% at a height of 1 mm from the top of the crest, 58.8% at 3 mm height, 46.7% at 5 mm (p≤0.05). Certain patterns of bone tissue growth were revealed depending on the structure of the reconstructed area. The most significant results were obtained in the molar segments of the mandible.

**Conclusion.:**

The developed modified two-stage split technique for alveolar ridge augmentation allows achieving the required volume of bone tissue in the posterior mandible for successful implant treatment.

## Introduction

Today, rehabilitation of patients using dental implants in conditions of atrophic jawbone is one of the common problems of dentistry [1]. Intensity and severity of destructive processes in the alveolar portions of the jaws results from various pathological conditions of the maxillofacial region: congenital pathologies; periodontal diseases, especially those with rapidly progressive forms; traumatic tooth extraction ignorant of the manipulation technique or in root fracture development; traumatic injuries; oncological resection of bone tissue; long-term usage of a removable denture [2].

However, bone deficiency also occurs as a physiological process associated with tooth loss. The alveolar bone, including the bundle bone, is closely connected with the periodontium, which is determined by the unity of their development, structure, and function. Damage to the periodontal tissue during tooth extraction inevitably initiates bone tissue atrophy as a physiological reaction in response to the impairment of functional-anatomical relationship between the periodontium and the alveolar walls and impaired blood supply in this area.

First of all, the vestibular alveolar bone wall undergoes resorption, especially, if it has insignificant thickness [3]. Primary bone remodeling of the alveolar ridge occurs rapidly, and horizontal (sagittal) atrophy develops in a very short time (up to 3 months) [4], with bone loss up to 63% recorded after 6 months [5].

The presented problem is especially relevant for the posterior areas of the jaws. Patients often neglect this segment, since it represents no aesthetic value. They pay attention to the presence of a defect only when applying for masticatory function restoration or when feeling discomfort and pain in the temporomandibular joint [6]. This leads to gross impairment of morphological characteristics of the alveolar ridge continuing until it disappears, which requires the use of reconstructive technologies to enable adequate rational implant treatment.

To restore the functional balance of the dentofacial system and masticatory function, implants should be installed in the appropriate orthopedic position without being guided by the existing bone tissue volume. Therefore, rational orthopedic-oriented implant treatment is impossible without the use of reconstructive technologies aimed at restoring bone volume.

Currently, there are various methods for reconstructing the bone tissue of the edentulous alveolar ridge to eliminate horizontal atrophy: plastic surgery using autogenous bone blocks [7–12], guided bone regeneration [13–16], alveolar ridge spreading technique [17], alveolar ridge splitting technique [18–21], distraction osteogenesis [22]. Despite the large number of research papers in both domestic and foreign literature, there is an ongoing discussion about choosing the most optimal method for achieving this goal. Researchers in surgical implantology aim at developing and implementing new methods to restore the lost tissue and achieve predictable, stable, and long-term implant treatment results.

**The aim of the study** was to evaluate the results of the developed method for bone grafting, a modified two-stage split technique for controlled ridge augmentation in horizontally atrophic posterior mandible, using radiological analysis data.

## Materials and Methods

This study was carried out in accordance with the principles of the Helsinki Declaration (2013). All procedures and materials were approved by the Ethics Committee of Privolzhsky Research Medical University. Written informed consent was obtained from all patients prior to starting the work.

Examination and surgical intervention were carried out in 18 patients with horizontally atrophic posterior mandible at the Department of Oral and Maxillofacial Surgery with the Course of Plastic Surgery, Privolzhsky Research Medical University (Nizhny Novgorod). The study included only relatively healthy patients with adequate oral hygiene. Besides, a precondition for inclusion was smoking no more than 10 cigarettes per day. Most of those under study were females, the average age of the group was 46 years (27 to 65 years).

A total of 39 jaw segments were examined, in which there were 22% of single and 78% of multiple defects; 33% bounded and 67% distal edentulous fragments. According to the area of the mandible, the defects were distributed as follows: 9% were in the area of first premolars; 32% — second premolars; 41% — first molars; 18% — second molars.

All patients underwent a comprehensive examination, including general clinical examination and cone-beam computed tomography using the Vatech PaX-i 3D computed tomograph (Vatech, South Korea).

Analysis of computed tomograms of the jaws, visualization of the morphological features of the atrophic alveolar ridge structure and identification of anatomically important structures were used to establish the following essential conditions for inclusion in the study:

absence of vertical atrophy;

sufficient vertical size of the jaw (13 mm or more to the upper border of the mandibular canal);

the presence of a spongy layer between the vestibular and lingual cortical plates (at least 1 mm);

the alveolar ridge width at a distance of 3 mm from its top being less than 3 mm;

the time after tooth extraction being at least 5–6 months.

**Figure 1:**
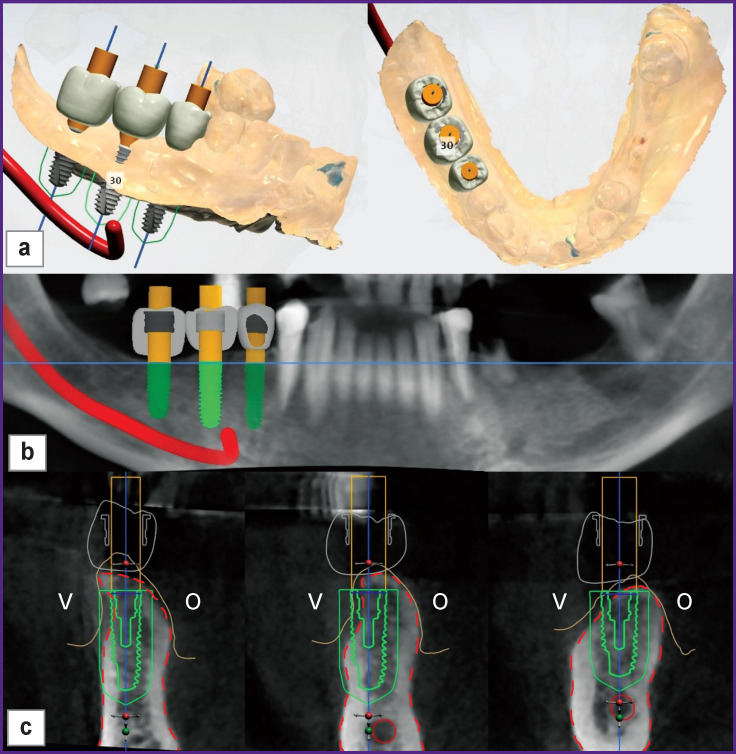
Virtual planning of implantation: (a) a three-dimensional image of the digital model; (b) panoramic view; (c) the image of the segments. Here: V — vestibular side; O — oral side; white — the supposed orthopedic structure; green — an implant with a safety zone surrounding it; the red solid line is the mandibular canal; the yellow line is mucous membrane profile; the dashed red line indicates bone tissue boundaries

Prior to surgery, virtual modeling of the proposed orthopedic structures and positioning of implants were performed using the Implant Studio software (3Shape, Denmark) (Figure 1). This preliminary planning of implant treatment made it possible to identify bone deficiency in the reconstructed area, determine the vertical axis of the implant, and the direction vector required for bone augmentation. Visualization of the future orthopedic structure allowed determining the vertical axis of the segment most accurately. Comparison of the selected axes on computed tomograms before and after reconstruction made it possible to clearly determine the identity of the studied areas and avoid errors in assessment of surgical treatment results.

**Figure 2:**
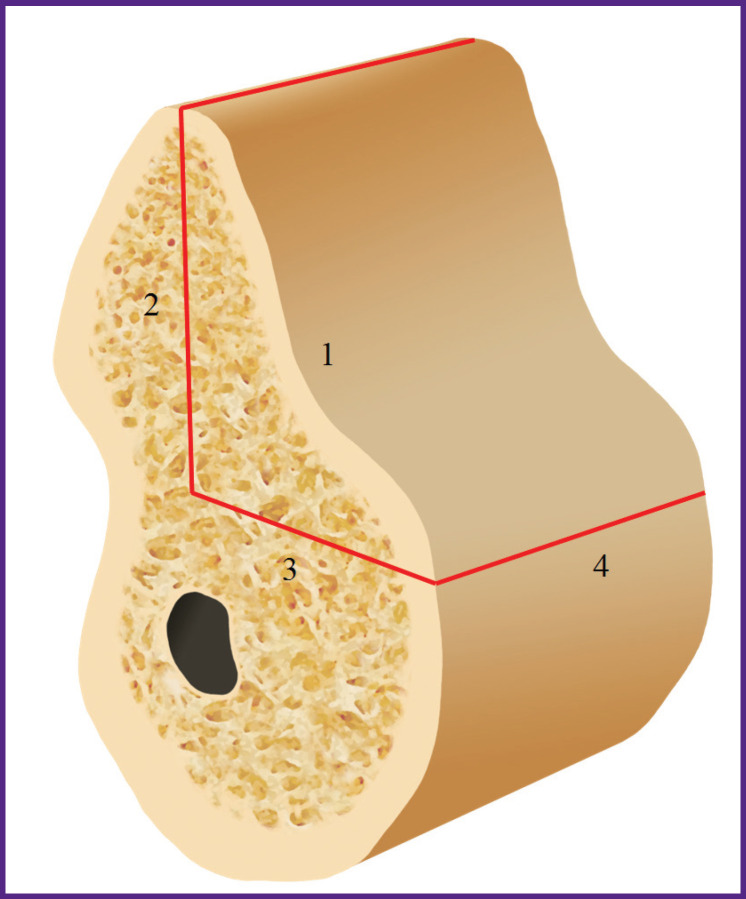
Osteotomy scheme: *1* — the vestibular wall, base of the triangle; *2* — the height of the osteotomy from the top of the alveolar ridge, the first leg; *3* — the depth of the horizontal apical osteotomy, the second leg; *4* — apical hinge osteotomy

**Figure 3:**
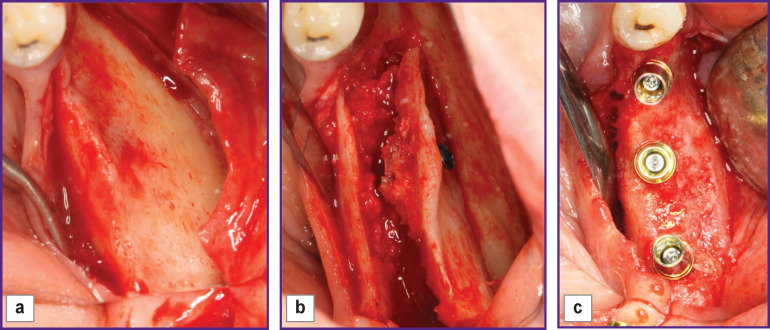
Clinical example of osteoplasty: (a) the lateral part of the mandible (sagittal atrophy of bone tissue); (b) modified split-technique; (c) augmented area of the mandible

All patients underwent bone graft surgery using the developed method of modified two-stage split technique for controlled ridge augmentation [6]. This method is characterized by making cuts with the formation of a mobilized osteotomized fragment of the vestibular bone plate (Figure 2). The sectional view of this fragment has the form of a triangle, whose base is the vestibular wall, one leg is the cut height from the ridge top, while the other leg is the depth of apical horizontal osteotomy. The osteotomized portion rotates on the apical hinge cut, the most convex part of the triangle moves from the basal to the middle part of the defect. The fragment is fixed in this position, which allows controllable augmentation of the alveolar part (Figure 3).

After 6 months following bone grafting, all patients underwent clinical examination (Figure 3 (c)) and repeated computed tomography.

**Figure 4:**
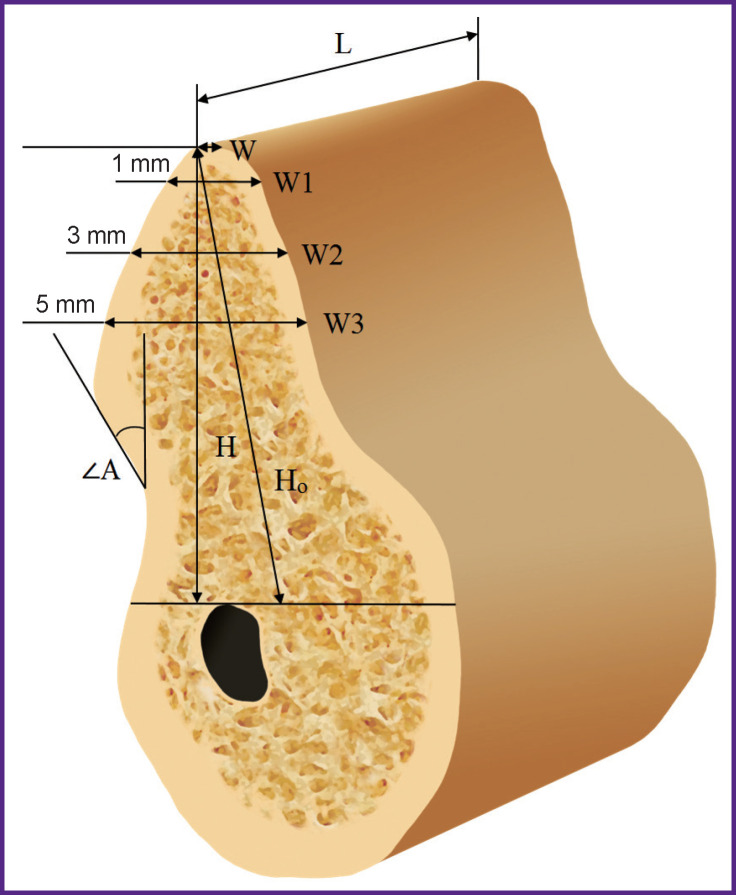
Scheme of measurements on cone-beam computed tomography: *H_o_* is the total height of the residual alveolar ridge along its length; *H* is the height of the vertical alveolar ridge; *L* is the length of the edentulous area; *W* is the alveolar ridge width at a height of 1 mm (*W*1), 3 mm (*W*2), 5 mm (*W*3) from its top; <*A* — angle of inclination of the alveolar ridge

EzDent software was used to carry out radiographic measurements on computed tomograms. Measurements were carried out before treatment and 6 months after bone grafting based on the same reference lines (Figure 4). The axes were positioned in the same way during both studies, relying on the structures remaining unchanged for 6 months (mandibular angle, mental foramen, mandibular canal, mental prominence).

The alveolar ridge was rotated so that the vertical axis was parallel to the axis of the intended implant position. Two horizontal reference lines perpendicular to the vertical were drawn: one along the top of the alveolar ridge, and the other along the upper border of the mandibular canal. The following parameters were measured:

total height of the residual alveolar ridge (*H_о_*) — the distance from the top of the ridge to the mandibular canal in the direction of the alveolar ridge (mm);

length of the edentulous space (*L*) — mesiodistal distance (mm);

the alveolar ridge height (*H*) in a vertical position, i.e. the shortest distance between two horizontal reference lines parallel to the orthopedic position of the implant (mm).

The height *H* was defined as the third reference line, relative to which the alveolar ridge width was measured at the top (*W*) and a height of 1, 3, 5 mm (*W*1, *W*2, *W*3, respectively). Width measurements were performed with the alveolar ridge in the vertical position corresponding to the expected position of the future implant.

Horizontal reference lines (without changing the distance between them) were transferred to computed tomography 6 months after bone grafting. This provided measuring the alveolar ridge width at the same levels, regardless of changes in *H*. Besides, the angle between the vertical and lingual inclination of the jaw (angle *A*) was measured, i.e. the angle of inclination of the alveolar ridge.

Measurements taken on control tomograms 6 months after the surgery were compared to the initial (before treatment) level of bone tissue, the difference and the percentage of increase in bone tissue after bone grafting were determined.

**Statistical data processing** was carried out using the Statistica 10.0 software package. The resulting data were presented as mean value (M) and standard deviation (σ). Reliability of the results was assessed using Student’s t-test for parametric research methods and small samples.

## Results and Discussion

Our measurements of the alveolar ridge width before treatment and 6 months after bone grafting with the use of two-stage split technique in the posterior mandible showed that the average increase in bone tissue (*W*) was 82% at the top of the alveolar ridge; 50.6% — at a height of 1 mm from the top (*W*1); 58.8% — at 3 mm (*W*2); 46.7% — at 5 mm (*W*3) (Table 1).

**Table 1:** The width of the alveolar part of the mandible in the reconstructed region (M±σ)

Parameter	Before treatment (mm)	6 months after bone grafting (mm)	Increase in bone tissue (%)
*W*	1.95±0.31	3.55±0.51*	82.05
*W*1	3.56±0.32	5.37±0.40*	50.60
*W*2	5.13±0.35	8.14±0.32*	58.81
*W*3	6.53±0.37	9.57±0.39*	46.67

* Statistically significant differences in values as compared to the results before treatment, p≤0.05.

However, it seemed reasonable to analyze the dynamics of the studied parameters for each segment (Table 2) due to the differences in the structure of the mandible (angle of inclination, the ratio of compact and spongy substance, the location of the mandibular canal) along the entire length of the edentulous area.

**Table 2:** The width of the alveolar part of the mandible in the reconstructed region in segments (M±σ)

Segment	The angle of inclination of the alveolar ridge (deg)	Width	Before treatment (mm)	6 months after bone grafting (mm)	Increase in bone tissue (%)
First premolars (3.4; 4.4)	11.62±1.93	*W*	1.50±0.20	3.45±0.75*	130.0
		*W*1	3.75±0.75	5.95±0.85	58.67
		*W*2	4.77±0.19	7.43±0.57*	55.95
		*W*3	5.83±0.56	8.80±0.40*	50.87
Second premolars (3.5; 4.5)	9.97±1.69	*W*	2.12±0.70	3.15±1.85	48.23
		*W*1	3.13±0.51	4.34±1.37	38.49
		*W*2	4.46±0.48	7.81±0.48*	75.32
		*W*3	5.80±0.48	9.09±0.45*	56.66
First molars (3.6; 4.6)	28.64±2.96	*W*	1.39±0.22	2.74±0.51*	97.62
		*W*1	2.69±0.36	5.30±0.68*	97.10
		*W*2	4.72±0.34	7.64±0.60*	61.88
		*W*3	6.44±0.39	9.21±0.61*	42.94
Second molars (3.7; 4.7)	42.25±3.83	*W*	1.82±0.36	4.30±0.13*	135.62
		*W*1	4.30±0.59	5.45±0.87	26.74
		*W*2	6.87±0.69	8.52±0.58	24.00
		*W*3	8.68±0.54	10.57±0.47*	21.90

* Statistically significant differences in values as compared to the results before treatment, p≤0.05.

The following data were obtained when studying the dynamics of parameters by segments in more detail. The average percentage of increase in bone tissue in the area of first premolars (3.4; 4.4) (angle *A* — 11.62±1.93º) was 130.0% at the top of the alveolar ridge; at a height of 1 mm from the top — 58.6%; 3 mm — 55.9%; 5 mm— 50.9%.

The average increase in bone tissue in the area of second premolars (3.5; 4.5) (angle *A* — 9.97±1.69º) was 48.2% at the top of the alveolar ridge; at a height of 1 mm from the top — 38.5%; 3 mm — 75.3%; 5 mm — 56.6%.

The average increase in bone width 6 months after bone grafting in the area of first molars (3.6; 4.6) (angle *A* — 28.64±2.96º) was 97.6% at the top of the alveolar ridge; 97% — at a height of 1 mm from the top; 61.9% — at 3 mm; 42.9% — at 5 mm.

**Figure 5:**
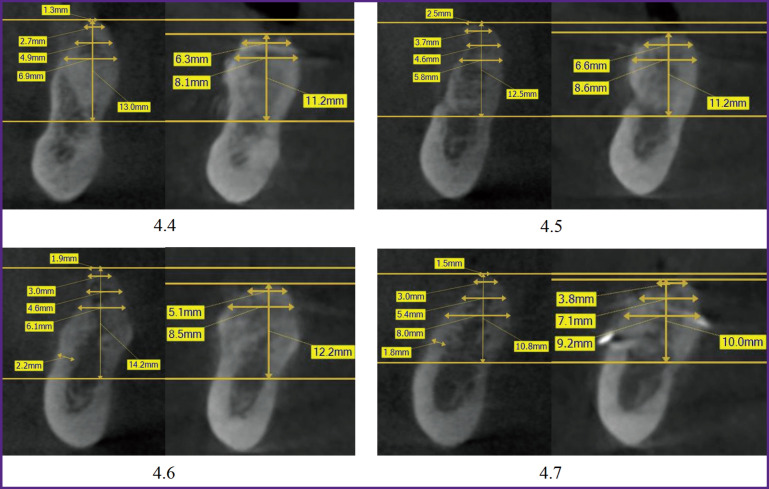
Measurements on cone-beam computed tomography before treatment (*left*) and 6 months after bone grafting (*right*) in the jaw segments

The average increase in bone tissue in the area of second molars (3.7; 4.7) (angle *A* — 42.25±3.83º) was 135.6% at the top of the alveolar ridge; 26.7% — at a height of 1 mm from the top; 24% — at 3 mm; 21.9% — at 5 mm (see Table 2, Figure 5).

Particular attention was paid to the comparative analysis of the alveolar ridge height in the bone reconstruction area as there are available literature data on the possible decrease in the vertical size of the bone tissue *H* after using alveolar ridge splitting techniques [23]: before treatment — 15.85±0.64 mm; 6 months after bone grafting — 15.59±0.70 mm.

According to our observations, this parameter remained unchanged 6 months after bone grafting in 43.48% of cases. There was an increase in bone tissue height by 0.720±0.174 mm (from 0.5 to 1.4 mm) in 21.74% of cases. In 34.78%, there was a decrease of 1.188±0.179 mm (from 0.6 to 2 mm) in bone tissue. A decrease in height was observed in such clinical cases when the top of the alveolar ridge had the minimal width initially, was represented by cortical bone tissue at a considerable distance and there was a low angle of mandibular inclination. In all cases, this was found in the area of mandibular premolars, which affected the overall calculation of width parameters at points *W* and *W*1.

As a rule, the analysis of changes in the alveolar ridge width is quite subjective even at 1 mm distance from it. Measurements at the points selected at a height of 3 and 5 mm were the most important, therefore, the average decrease in bone tissue height at a point of 1 mm was not crucial when choosing the implant diameter. Moreover, this decrease causes no disturbances in the relationship between implant length and crown height.

According to the results of analysis of the edentulous areas in the posterior mandible before treatment, we revealed a tendency for displacement of the top of the crest and rather a large angle of lingual inclination of the alveolar ridge in accordance with the anatomical structure of the mandible in the study area.

When the angle of lingual inclination of the alveolar ridge is more than 25º, bone grafting in the sagittal direction is required, despite the sufficient volume of bone tissue at the top of the edentulous ridge. This is necessary to avoid excessive lingual position of the implant platform and to prevent perforation of the lingual cortical plate. Perforation of the lingual wall can lead to its necrotization. Besides, it increases the risk of damage to the sublingual artery. The method of two-stage alveolar ridge splitting is a direct indication in the above clinical situations.

The data obtained demonstrate that the method of controlled ridge augmentation allows achieving the volume of bone tissue that ensures implant placement in the correct orthopedic position. During installation, implants were surrounded on both vestibular and oral sides by at least 1.5 mm bone tissue, which meets the basic implantation criteria [24, 25].

## Conclusion

Radiological analysis of the developed two-stage split-technique for controlled ridge augmentation in horizontally atrophic posterior mandible proved its efficacy. This method provides the possibility to achieve the required volume of bone tissue adequate for successful implant treatment in compliance with all modern recommendations for placing implants in the correct orthopedic position. We managed to achieve the most significant changes in the alveolar part of the mandible in the area of molar segments, both in terms of absolute values and in the vestibular displacement of the plane of future implant location.

However, objective assessment of any jaw-bone reconstruction method efficacy is impossible without joint analysis of clinical intraoperative and radiological examination results with due regard for the histological characteristics of regeneration area, which is most likely to increase predictability of surgical intervention.
